# Short-term trends in hypertension and high cardiovascular risk at high altitude in Peru

**DOI:** 10.3389/fpubh.2025.1704843

**Published:** 2026-01-30

**Authors:** Brando Ortiz-Saavedra, Julio S. Mamani-Castillo, Irmia Paz, Jorge Ballón-Echegaray, Ricardo A. J. Leon-Vasquez

**Affiliations:** Universidad Nacional de San Agustín de Arequipa, Arequipa, Peru

**Keywords:** hypertension, cardiovascular risk, high-altitude, trends, Peru

## Abstract

**Objective:**

The aim of the present study was to describe short-term trends in the prevalence of high cardiovascular risk and the prevalence, awareness, treatment, and controlled hypertension in the high altitude of Peru, during the years 2017 to 2023, using data from the Demographic and Family Health Survey.

**Methods:**

A cross-sectional analysis was carried out using secondary data of six representative surveys nationwide (2017–2023, excluding the 2020 survey). For the analysis of hypertension and cardiovascular risk, we included 56,404 and 38,221 participants residing in high-altitude areas, respectively. The mean and age-standardized prevalence were calculated with their respective 95% confidence intervals and the trends of the estimated proportions were evaluated with Mann-Kendall test. A value *p* < 0.05 statistically significant was considered.

**Results:**

An increase in the age-standardized prevalence of hypertension, disease knowledge, treatment, and controlled hypertension was identified between 2017 and 2023; however, no statistically significant trends were observed in these prevalences. The proportion of participants with high cardiovascular risk remained constant during this period.

**Conclusion:**

In participants residing in high-altitude cities in Peru, no statistically significant trends were found in the age-standardized prevalences of hypertension, disease knowledge, treatment, controlled hypertension, or cardiovascular risk between 2017 and 2023.

## 1 Introduction

Hypertension is a leading cause of premature death worldwide and is a major contributor to the global burden of disease (1, 2). The World Health Organization (WHO) estimates that 1.28 billion adults aged 30–79 years worldwide have hypertension, two-thirds of whom live in low- and middle-income countries (LMICs) (2). In these countries, it has been reported that despite a high prevalence of hypertension, there is low awareness of hypertension and therefore a low proportion of adults with hypertension receive adequate treatment (3). In Peru, the mortality rate from hypertensive diseases increased, rising from 15.2 deaths per 100,000 inhabitants (2017) to 38.5 deaths per 100,000 inhabitants (2021) (4). Hypertension is the most important risk factor for the development of cardiovascular disease (CVD). The highest rates of CVD morbidity and mortality are found in LMICs, where approximately more than 80% of CVD deaths occur and 40% of these deaths are premature (before the age of 70 years), well above those in high-income countries (15%) (5). In Peru, approximately 15% of all causes of premature death are caused by CVD, with a mortality rate of 143 deaths per 100000 inhabitants (6).

Villareal-Zegarra et al. (3) performed an analysis in Peru using representative surveys during the period 2015 to 2018 and found that an increase in the age-standardized prevalence of hypertension and a decrease in the prevalence of people with disease awareness and controlled hypertension. However, they did not perform a sub analysis for the population living at high altitude. Peru is a country in the Andean region of South America, where approximately 30% of its population lives at high altitude (> 2500 meters above sea level (masl)) and most of its high-altitude cities are rural areas. The high-altitude environment is characterized by a low partial pressure of O2 due to decreased barometric pressure (hypobaric hypoxia) and long-term exposure to this condition could be associated with a lower incidence of hypertension and CVD in its residents, since there would be complex interactions between individual physiological and behavioral characteristics with environmental ones (7, 8). These particularities could change the trends in the prevalence of hypertension and high cardiovascular risk. Furthermore, identifying current trends in hypertension awareness, treatment, and control allows the development of policies and interventions aimed at better resource allocation in LMICs (3). Therefore, the objective of the present study is to describe short-term trends in the prevalence of high cardiovascular risk and the prevalence, awareness, treatment, and controlled hypertension in the high altitude of Peru, during the years 2017 to 2023, using data from the Demographic and Family Health Survey (ENDES).

## 2 Methods

### 2.1 Study design, data source, and sampling

ENDES is a cross-sectional survey with national, regional and urban–rural representativeness and is conducted annually according to the guidelines of the Demographic and Health Survey Program (DHS). The sample is two-stage, probabilistic, balanced, stratified and independent. Initially, the Primary Sampling Unit (PSU) was selected based on its weight in occupied dwellings in accordance with the balanced sampling of the framework of the last national census of 2017. Subsequently, the Secondary Sampling Unit (SSU) was selected considering the previously framed target population (children under 5 years of age, women of childbearing age, etc.). The primary method used for data collection was face-to-face interviews, conducted by interviewers trained in filling out the forms, who visited each SSU to complete the designated questionnaires (9). In the analysis, surveys carried out from 2017 to 2023 were included, and the 2020 survey were excluded because it had a large amount of missing data. Data from the ENDES surveys are available online.^1^ No ethics committee approval was required since this was a secondary analysis of public domain data (9).

### 2.2 Variables

#### 2.2.1 Hypertension

Hypertension was defined as Systolic Blood Pressure ≥140 mmHg (SBP) or Diastolic Blood Pressure ≥90 mmHg (DBP) (average of 2 blood pressure measurements) or if participants had a previous diagnosis of hypertension (3, 7). Blood pressure was measured using a validated digital blood pressure monitor by a previously trained interviewer, supervised by an anthropometrist who was responsible for supervising and explaining the results to the respondent. Participants were asked whether they had consumed tea, coffee, hot beverages, alcohol, or smoked in the previous 30 min before the blood pressure measurement; if so, a 30-min wait was required between consumption and measurement. After the first measurement with the digital blood pressure monitor, a 2-min wait was required before the second measurement was taken on the same arm (10).

#### 2.2.2 Disease awareness

Participants with disease awareness were those who reported having received a previous diagnosis of hypertension.

#### 2.2.3 Hypertensive patients with treatment

These were participants who were aware of their hypertension diagnosis and who reported taking prescribed antihypertensive medication.

#### 2.2.4 Patients with controlled hypertension

Hypertensive participants with awareness of their disease, who reported taking their prescribed medication and who have SBP < 140 and DBP < 90 for those under 60 years of age and SBP < 150 and DBP < 90 for participants aged 60 years or older (11).

### 2.3 Cardiovascular risk

The *CVrisk* command, using the non-laboratorial Framingham risk score, calculates the estimated 10-year risk of atherosclerotic cardiovascular disease. It uses the parameters sex, age between 30 and 74 years, body mass index (BMI), SBP, whether the patient is taking blood pressure medication, whether he or she is a current smoker, and whether he or she has diabetes. A score >20% was classified as high risk, a 10–20% score was classified as intermediate, and a < 10% score was classified as low risk (12).

### 2.4 Selection criteria

For the analysis required for hypertension, participants were included 18 years or older, while for the calculation of cardiovascular risk, only participants from 30 to 74 years were included. Only participants who resided in households located at> 2500 masl were included. For hypertension analysis, participants were excluded who had incomplete blood pressure data or who had biologically unlikely measurements (SBP > 270 mmhg or <70, and DBP > 150 mmhg or <50). For the calculation of cardiovascular risk, participants with incomplete data for each of the variables required by the *CVRisk* command were excluded. With respect to BMI, participants with biologically unlikely BMI measurements (BMI > 60 kg/m^2^ or < 10 kg/m^2^) were excluded (7).

### 2.5 Statistical analysis

Statistical analysis was performed in R (version 4.0.2). In all analyses, the sample design was specified. We calculated mean and weighted proportions with their respective 95% confidence intervals (95% CI). Age-standardized prevalence were calculated (13) and the Mann-Kendall test was applied to assess trends according to prevalence (14). We considered the *p*-value <0.05 to be statistically significant. We also performed a descriptive analysis for participants living at low altitude, which can be found in the Supplementary Material.

## 3 Results

### 3.1 Participants

**Figure 1:**
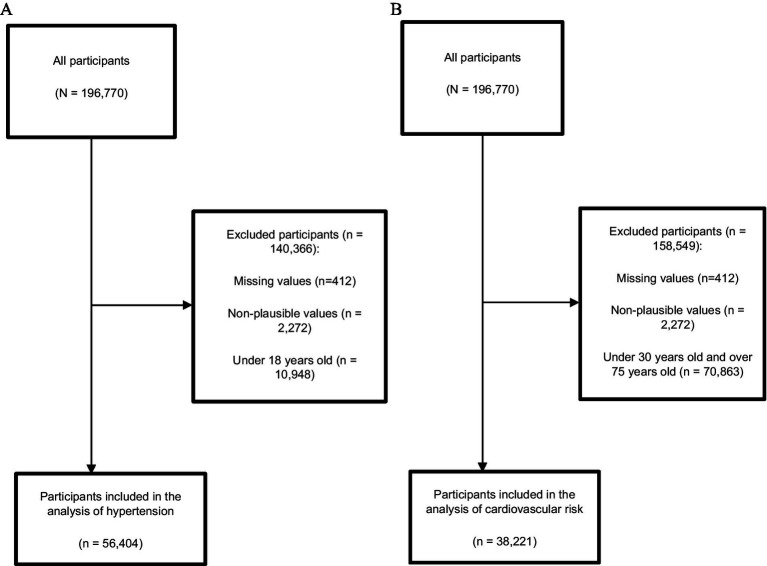
Participant selection flowchart **(A)** Hypertension analysis, **(B)** Cardiovascular risk analysis.

Initially, a total of 196,770 participants were included between 2017 and 2023 (excluding 2020), and 140,366 participants were excluded. Details are provided in Figure 1. For the hypertension analysis, a total of 56,404 participants living at high altitude were included. For the cardiovascular risk analysis, 38,221 participants living at high altitude were included (Figure 1B).Table 1 describes the main characteristics according to the years studied. Of the total participants included, the majority were aged 18 to 44 years (57.0%), female (53.4%) and resided in urban areas (52.7%).

**Table 1:** Characteristics of participants (*N* = 56,404).

Characteristics of participants by year	2017		2018		2019		2021		2022		2023		Total	
	%	95% CI	%	95% CI	%	95% CI	%	95% CI	%	95% CI	%	95% CI	%	95% CI
Age														
18–44	57.8	56.2–59.4	56.7	55.2–58.0	56.2	54.8–57.5	57.8	56.2–59.3	57.1	55.7–58.5	56.7	55.1–58.2	57	(56.4–57.6)
45–54	16.9	15.8–18.0	17.1	16.1–18.2	17	15.8–18.3	16.4	15.3–17.6	16.7	15.6–17.9	16.6	15.4–17.8	16.8	(16.3–17.3)
55–64	10.8	10.0–11.7	11.4	10.5–12.2	11.8	11.0–12.6	11.7	10.8–12.7	12.1	11.2–13.2	12.6	11.7–13.7	11.7	(11.4–12.1)
65–74	8	7.3–8.8	8.5	7.8–9.3	8.4	7.7–9.2	7.9	7.1–8.8	8.1	7.3–9.0	8.2	7.4–9.1	8.2	(7.9–8.5)
>74	6.5	5.8–7.2	6.4	5.6–7.2	6.6	5.9–7.4	6.1	5.4–6.9	5.9	5.2–6.7	5.9	5.2–6.8	6.2	(5.9–6.5)
Sex														
Female	52.9	51.6–54.3	52.8	51.3–54.2	53.8	52.3–55.2	53.3	51.9–54.7	54.2	52.8–55.7	53.4	51.9–54.9	53.4	(52.8–54.0)
Male	47.1	45.7–48.4	47.2	45.8–48.7	46.2	44.8–47.7	46.7	45.3–48.1	45.8	44.3–47.2	46.6	45.1–48.1	46.6	(46.0–47.2)
Area of residence														
Urban	48.2	44.7–51.8	51.9	49.5–54.2	52.7	50.3–55.0	53.5	51.0–55.9	54	51.7–56.3	56.2	53.7–58.7	52.7	(51.7–53.8)
Rural	51.8	48.2–55.3	48.1	45.8–50.5	47.3	45.0–49.7	46.5	44.1–49.0	46	43.7–48.3	43.8	41.3–46.3	47.3	(46.2–48.3)

### 3.2 Trends for hypertension

**Figure 2:**
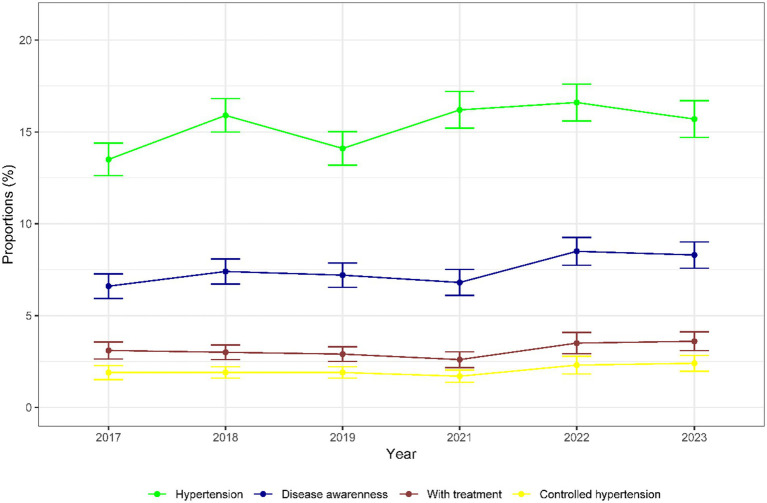
Prevalence of hypertensive patients, disease awareness, with treatment, and controlled hypertension (2017–2023).

Figure 2 shows the trends described for each year. The age-standardized prevalence of hypertension at high altitude increased from 13.5% (95% CI: 12.6–14.4) by 2017 to 15.7% (95% CI: 14.7–16.7) by 2023, although there was no significant increase in trend (*p* = 0.26). There was an increase in prevalence of 2.1% between 2019 (14.1%) and 2021 (16.2%). Regarding disease awareness, prevalence increased from 6.6% (95% CI: 5.9–7.3) by 2017 to 8.3% (95% CI: 7.6–9.0) by 2023, but there was no significant increase in trend (p = 0.26). Likewise, the proportion of participants with hypertension with treatment increased by 0.5%, being 3.1% (95% CI: 2.6–3.6) by 2017 and 3.6% (95% CI: 3.1–4.1) by 2023, however there was no significant increase in trend (*p* = 0.71). Finally, the proportion of participants with controlled hypertension also increased 0.5%, being 1.9% (95% CI: 1.5–2.3) by 2017 and 2.4% (95% CI: 2.0–2.8) by 2023, but there was no significant increase in trend (*p* = 0.32). For the proportions of participants with diseases awareness, treatment, and controlled hypertension, there was a slight decrease from 2019 to 2021 (0.4, 0.3, and 0.2%, respectively).

### 3.3 Trends for cardiovascular risk

**Figure 3:**
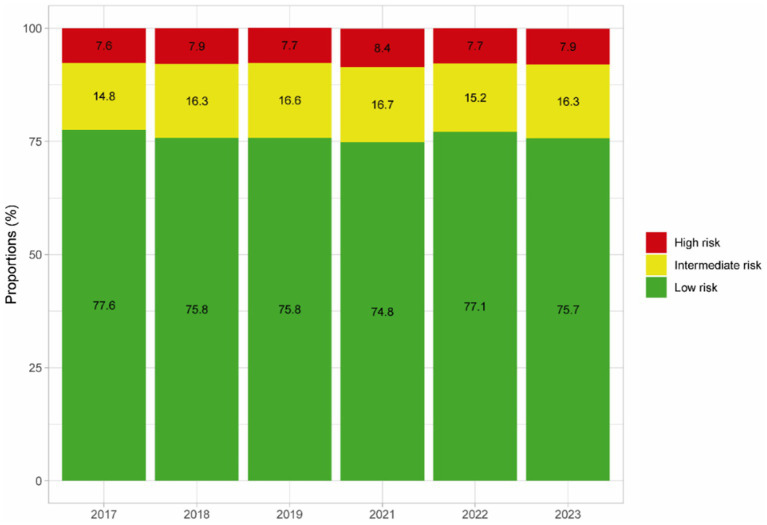
Cardiovascular risk for each year (2017–2023).

The mean cardiovascular risk at high altitude was 7.28 (95% CI: 7.17–7.38). The proportion of high cardiovascular risk in all participants included was 7.9% (95% CI: 7.5–8.3). Figure 3 shows the proportion of cardiovascular risk for each year categorized as low, intermediate, and high. The prevalence of high risk remained with small changes over time, being 7.6% (95% CI: 6.7–8.5) for the year 2017 and 7.9% (95% CI: 6.9–9.1) for the year 2023 (*p* = 0.85).

## 4 Discussion

### 4.1 Main findings

The main objective of the present study was to describe short-term trends in the prevalence of high cardiovascular risk and the prevalence, awareness, treatment, and controlled hypertension in the high altitude of Peru, during the years 2017 to 2023. Although during the period from 2017 to 2023, the prevalence of hypertension, diseases awareness, treatment, and controlled hypertension increased, the trends were not significant. Likewise, the proportion of people with high cardiovascular risk remained constant during this period.

### 4.2 Comparison with previous studies

The age-standardized prevalence of hypertension we found in 2023 in people living at high altitude was 15.7%. Our results agree with those previously reported in local studies. In the study by Zila-Velasquez et al. (15), they meta-analyzed results from 11 studies conducted in Peru and found that the prevalence of hypertension at altitude (>1500 masl) was 14.6% (95% CI: 11.5–17.8) for a cutoff point of ≥140/90 mmHg. Similarly, two studies conducted in Peru (not included in the above meta-analysis), found a prevalence of 16.0% in cities located between 2500 and 3499 masl (16) and 17.0% in cities located at > 2500 masl (7). However, the reported prevalence is lower compared to those reported in other Andean countries. In a study conducted in Ecuador, in people residing at an average altitude of 2800 masl, the prevalence of hypertension was found to be 27% (17). Furthermore, in a study carried out in Colombia, they found that the prevalence of hypertension was 36% in people living at more than 2000 masl (18). The results we found also differ from those found in other communities living at high altitude, such as on the Tibetan plateau. In a study by Zheng et al. (19) in 1416 participants residing in the city of Lhasa, Tibet, located at 3650 masl on average, they found that the prevalence of hypertension was 51.2%. Also, Li et al. (20) conducted a study in prefecture of Ngawa, Tibet, where they included 2,228 participants, and found that the prevalence of hypertension was 18.4% in cities located between 2500 and 3500 masl, however, the prevalence increased in cities located at ≥3500 masl, being 34.4%.

We also found that the mean cardiovascular risk at high altitude was 7.28 and that there were no significant changes in the trends for the proportions at high cardiovascular risk. These results contrast with those previously reported in Peru. Hernández-Vásquez et al. (8) performed an analysis of 833 participants aged 18 to 59 years from a nationally representative sample, and reported a mean cardiovascular risk score of 3.4 in persons residing at ≥2500 masl. Likewise, Bernabe-Ortiz et al. (21) performed an analysis of several ENDES, including a total of 80,409 participants aged 40 to 79 years, and found a mean score of 4.5 and that only 7.8% of participants had a cardiovascular risk ≥10% in the general population; however, they did not report results according to altitude. It is important to comment that both studies used different scores than the one used in the present study.

In Peru, there was a progressive increase in the prevalence of hypertension. This increase could be due to the epidemiological transition caused by continued economic growth and exposure to risk factors (such as sedentary lifestyles and smoking) (3). Although several studies have found that the prevalence of hypertension is lower in people living at high altitude in Peru (7, 16), it is necessary to propose and reinforce prevention, diagnosis and treatment initiatives in health facilities located at high altitude, considering the particular environmental, physiological and behavioral characteristics (dietary habits and physical activity) of these regions, in order to avoid a constant increase in the trends found in the present study.

### 4.3 Limitations and strengths

The present study has certain limitations. First, ENDES collected two blood pressure measurements, which could result in a misclassification of hypertension; however, previous studies considered other criteria for a correct detection of hypertension, obtaining similar results (3, 7, 16). Second, the phenotype of masked hypertension that is very prevalent in high-altitude areas of Peru was not considered, which could underestimate the real prevalence of hypertension (22). Third, recall bias could be present in the reported author variables of previous diagnosis of hypertension. Fourth, ENDES did not measure other relevant variables that could support further understanding of the results, such as treatment adherence, drug treatment received, ethnicity, length of residence in the surveyed household, and migration to multiple households at different altitudes. While the strengths of the study lie in the fact that it included data from nationally representative surveys, the inclusion of a considerable amount of data, and that the team of surveyors had standardized training for blood pressure measurement with previously validated equipment (9).

## 5 Conclusion

In participants living in high-altitude cities in Peru, although an increase in the age-standardized prevalence of hypertension, awareness disease, treatment, and controlled hypertension was identified between 2017 and 2023, no statistically significant trend was found during the period from 2017 to 2023.

## Data Availability

The original contributions presented in the study are included in the article/Supplementary material, further inquiries can be directed to the corresponding authors.

## Footnotes

1
https://proyectos.inei.gob.pe/microdatos/
