# Supplementary material for: Short-term trends in hypertension and high cardiovascular risk at high altitude in Peru
Source: Front Public Health. 2026 Jan 30;13:1704843. doi: 10.3389/fpubh.2025.1704843 (PMC12901356; doi:10.3389/fpubh.2025.1704843)
Supplement: Supplementary file 1 [file Table_1.docx]

Supplementary Material

**Table S1.** Prevalence of hypertensive patients, knowledge of the disease, treatment and controlled hypertension stratified by area of ​​residence (high altitude).

| **Year** | **Condition** | **High Altitude** |
| --- | --- | --- |
|  |  | **Weighted proportion (CI 95%)** |
| 2017 | Hypertension | 13.5 (12.6-14.4) |
| 2018 | Hypertension | 15.9 (15-16.8) |
| 2019 | Hypertension | 14.1 (13.2-15) |
| 2021 | Hypertension | 16.2 (15.2-17.2) |
| 2022 | Hypertension | 16.6 (15.6-17.6) |
| 2023 | Hypertension | 15.7 (14.7-16.7) |
| 2017 | Disease awareness | 6.6 (5.9-7.3) |
| 2018 | Disease awareness | 7.4 (6.7-8.1) |
| 2019 | Disease awareness | 7.2 (6.5-7.9) |
| 2021 | Disease awareness | 6.8 (6.1-7.5) |
| 2022 | Disease awareness | 8.5 (7.7-9.3) |
| 2023 | Disease awareness | 8.3 (7.6-9) |
| 2017 | With treatment | 3.1 (2.6-3.6) |
| 2018 | With treatment | 3 (2.6-3.4) |
| 2019 | With treatment | 2.9 (2.5-3.3) |
| 2021 | With treatment | 2.6 (2.2-3) |
| 2022 | With treatment | 3.5 (2.9-4.1) |
| 2023 | With treatment | 3.6 (3.1-4.1) |
| 2017 | Controlled hypertension | 1.9 (1.5-2.3) |
| 2018 | Controlled hypertension | 1.9 (1.6-2.2) |
| 2019 | Controlled hypertension | 1.9 (1.6-2.2) |
| 2021 | Controlled hypertension | 1.7 (1.4-2) |
| 2022 | Controlled hypertension | 2.3 (1.8-2.8) |
| 2023 | Controlled hypertension | 2.4 (2-2.8) |

**Table S2.** Cardiovascular risk for each year stratified by area of ​​residence (high altitude).

| **Year** | **Risk** | **High Altitude** |
| --- | --- | --- |
|  |  | **Weighted proportion (CI 95%)** |
| 2017 | Low risk | 77.6 (76.1-79) |
| 2018 | Low risk | 75.8 (74.3-77.2) |
| 2019 | Low risk | 75.8 (74.2-77.2) |
| 2021 | Low risk | 74.8 (73.1-76.5) |
| 2022 | Low risk | 77.1 (75.4-78.7) |
| 2023 | Low risk | 75.7 (74.1-77.3) |
| 2017 | Intermediate risk | 14.8 (13.6-16.1) |
| 2018 | Intermediate risk | 16.3 (15.1-17.6) |
| 2019 | Intermediate risk | 16.6 (15.2-18.1) |
| 2021 | Intermediate risk | 16.7 (15.4-18.2) |
| 2022 | Intermediate risk | 15.2 (13.9-16.6) |
| 2023 | Intermediate risk | 16.3 (15-17.8) |
| 2017 | High risk | 7.6 (6.7-8.5) |
| 2018 | High risk | 7.9 (7-9) |
| 2019 | High risk | 7.7 (6.8-8.6) |
| 2021 | High risk | 8.4 (7.3-9.7) |
| 2022 | High risk | 7.7 (6.7-8.9) |
| 2023 | High risk | 7.9 (6.9-9.1) |
